# Effectiveness of Augmented Reality in the Teaching of Health University Students: Quasi-Experimental Study

**DOI:** 10.2196/54312

**Published:** 2025-03-27

**Authors:** Rocío Martín-Valero, Alejandro Vega-Morales Sr, Francisco Javier Martín-Vega, Manuel Rodriguez-Huguet, Maria Carmen Rodríguez-Martínez, Maria Jesus Vinolo-Gil

**Affiliations:** 1 Department of Physiotherapy, Faculty of Health Science, University of Málaga, Arquitecto Francisco Peñalosa 3, Ampliación de Campus de Teatinos, CTS-1071 Research Group, Málaga, 29071, Spain, 34 951952858; 2 Department of Nursing and Physiotherapy, University of Cádiz, Cádiz, Spain; 3 Biomedical Research Institute of Malaga-Nanomedicine Platform (IBIMA-BIONAND Platform), Málaga, Spain

**Keywords:** augmented reality, qualifications, usability, university, teaching, education, implementation, academic performance, quasi-experimental design, control group, applications, experimental group

## Abstract

**Background:**

The exponential growth of new technologies has resulted in the need for updating the field of education. From the educational point of view, there are some studies that have promoted the implementation of new technologies. These facts have raised the need to implement augmented reality in the university environment, especially among students of health sciences. The use of augmented reality can mean a new approach to teaching by teachers and better learning by students.

**Objective:**

We aimed to analyze the degree of usability of two augmented reality applications and to compare the academic performance between the control group and the experimental group at the Universities of Cádiz and Málaga. The students at the University of Málaga used the Zapworks augmented reality software, while those at the University of Cádiz used the Aumentaty augmented reality software for their respective experimental groups. The secondary objective was to measure the relationships between all the studied variables.

**Methods:**

This was a quasi-experimental design with a posttest as the only evaluation measure. We followed the SPIRIT (Standard Protocol Items: Recommendations for Interventional Trials) statement and the ethical and legal aspects of the Principles of the Declaration of Helsinki. An intervention was carried out using two augmented reality applications on the subject of General Procedures in Physiotherapy II at the Universities of Málaga and Cádiz.

**Results:**

A total of 199 participants took part in the study. Demographic variables, ratings, and usability were assessed, followed by statistical analysis, with the results and their interpretation being described in the study. Significant differences (*P*<.001) were found in the ratings at both the universities. In addition, significant differences (*P*<.001) were found between the experimental group and the control group. Regarding the degree of usability in the univariate analysis, no significant differences were found (*P*=.049). A multiple regression analysis of the rating and usability was performed. The rating showed significant differences, with a beta of 1.4 (*P*<.001), and usability was also significant (*P*=.03) in favor of the Aumentaty group.

**Conclusions:**

Significant differences were observed in those who used augmented reality compared to the control group, with higher values observed in the University of Cádiz. There are no correlations between the variables of usability and qualifications.

## Introduction

Currently, there is exponential development of new technologies in all areas (education, work, hospitals, etc). Therefore, given this obvious technological development, it is necessary to cover the needs that arise in the areas where they are being implemented, especially in the field of education [1]. New technologies such as computers, telecommunications, and all such devices and materials that enable advances in computer-mediated communication have not only exponentially broadened and improved our ability of handling, processing, and communicating information and knowledge on their own, but have also assimilated all the other resources that are now integrated into them [2].

Education must not remain on the side lines considering the evolution of new technologies, as they are a part of the basis of today’s society. Moreover, education is characterized by the use of the media employed in social communication, which are currently the new technologies [3]. Therefore, there is a need to investigate the use of new technologies in the field of education in order to analyze their use and effectiveness and therefore be able to implement new tools in the university education system.

Augmented reality (AR), understood as the combination of digital and physical information in real time through different technological devices, is becoming one of the emerging technologies with the closest penetration in university studies [4]. AR combines real and virtual objects in a real environment, aligns them together, and operates interactively in real time. It encompasses various senses beyond sight and is not limited to specific display technologies [5]. Additionally, AR includes the concept of overlaying virtual objects to remove or diminish real-world elements, known as mediated or diminished reality [6]. AR is presented as an attractive technology that promises to offer the tools needed to create engaging and motivating content [7-9]. Moreover, AR devices show potential in alleviating adverse health effects like blurred vision, disorientation, and cybersickness typically associated with prolonged utilization of virtual reality [10]. This allows different generic and transversal competences within the university environment to become effective. Furthermore, the use of this type of technology leads to a significant increase in motivation levels [11-14].

In a previous meta-review, the positive effects mentioned were enhanced comprehension of content, acquisition of spatial and language skills, improved long-term memory retention, and increased collaboration and motivation, while the negative effects included encompass narrowed attention, challenges in usability, ineffective integration in the classroom setting, and individual learner variations [15]. The importance of acquiring new abilities, working in enriched and motivating environments and, especially, assessing the possible usefulness of this tool in the performance of university students makes us reflect on the relevance of doing research on this aspect [11]. The significance has been explored in the field of health care, where some studies [16,17] have investigated the use of AR glasses for simulating venous catheter procedures in medical students. Furthermore, Logishetty et al [18] recognized AR as a valuable tool for other surgical procedures related to arthroplasty, as well as for novice ophthalmology [19]. Additionally, Barteit et al [20] presented the notion that incorporating virtual or AR teaching methods in medical education had advantageous outcomes, including heightened enthusiasm and enjoyment for learners.

For physiotherapy students, acquiring foundational knowledge and specific skills in subjects like body structure, biological functions, movement analysis, and motion characteristics through traditional teaching methods may be insufficient [21]. Moreover, it is crucial for them to acquire a diverse range of specialized competencies prior to commencing their professional journey in health care environments [22].

Mastering these skills demands the integration of intricate elements, hypothesis generation, analytical reasoning, and more. Traditional instructional approaches such as lectures, case study demonstrations, tutorials, and practical classes may present challenges in effectively acquiring these proficiencies [23]. However, research has demonstrated that incorporating audio-visual materials can enhance physiotherapy students’ enthusiasm for learning while also improving their grasp of theoretical concepts and practical aptitude [24]. Therefore, the use of AR technologies in physiotherapy education holds the potential for addressing challenges and providing innovative ways to enhance learning experiences for students [25-27].

In order to effectively integrate AR into education, it is crucial to design the teaching and learning process to fit the context. Teachers play a vital role in maximizing its benefits by taking on roles as planners, designers, and facilitators of the learning process. Additionally, when designing AR objects, the modules, content, and activities should be concise, interactive, and practical, allowing for student participation and action. The methodology should prioritize interactivity and practicality [28]. Regarding the types of AR according to the classification by Wojciechowski et al [29], it can be categorized into marker-based AR, markerless AR, and location-based AR. Marker-based AR is the most commonly used type (59.3%) in educational settings due to its superior tracking capabilities provided by markers. Marker-less AR, although less prevalent (12.5%), has potential with technologies like Microsoft Kinect for object tracking [30].

Moreover, it is worth noting that there are different software programs that can be used for AR. For the execution of this quasi-experimental design, two AR applications, common in teaching, were used. These applications are Zapworks and Aumentaty. Both types of AR utilize markers. There are other AR studies that address the importance of AR in the university environment within the field of health sciences; in the majority of them, the most important variable was the understanding of knowledge [7,31-33] together with motivation [7,33], qualifications [31,33], and attention and training [7,33]. Other variables considered were the autonomous work provided to students [7,33] and the assessment and use of new technologies [32]. One of the studies used Aumentaty as an AR tool in the same way as it was used in our design [31].

Zapworks is a software that allows the generation and delivery of digital content, which has made the use of AR more accessible. In contrast, Aumentaty is a platform focused on AR projects that allow the possibility of creating this type of technology in a simple way [34,35].

The aim was to compare the degree of usability of the two AR applications mentioned above. Moreover, the purpose was to compare the academic performance of the control group and the experimental group of the Universities of Cádiz and Málaga as a result of the use of the AR applications. Therefore, the null hypothesis will be the presence of significant differences between the control group and the experimental group in the variables of rating and usability, and the alternative hypothesis would be the absence of significant differences.

## Methods

### Study Design and Participants

This research was conducted through a quasi-experimental design with a posttest as the only evaluation measure. The design followed the SPIRIT (Standard Protocol Items: Recommendations for Interventional Trials) declaration where the protocols to be followed were established. The initial sample consisted of 203 students of health sciences who were enrolled in the Physiotherapy degree course from the Universities of Málaga and Cádiz. However, 4 participants over the age of 30 years were excluded from the analysis to homogenize the study. The inclusion criteria were students who were attending the Physiotherapy degree course and were enrolled in the subject of General Procedures in Physiotherapy II. The results of the study were measured in the 2021-2022 academic year.

### Ethical Considerations

The legal ethical aspects followed the principles of the World Medial Association and the Declaration of Helsinki for ensuring the principles and well-being of the study participants. Before the execution of the research intervention, participants signed an informed consent form in which they were provided with detailed information about the research. Their confidentiality and data protection were guaranteed at all times. Ethical approval for this educational research was obtained from the Andalusian Human Research Ethics Committee (approval number 001/2022). In addition, this study was conducted according to the ethical guidelines for educational research [36]. Participants in this research did not receive compensation.

### Study Procedures

The study was carried out in the subject of General Procedures in Physiotherapy II at the Universities of Málaga and Cádiz. Each teacher in charge of the subject explained to the students the possibility of participation in the study and, therefore, the use of the AR applications, with which the general and transversal competences of the subject would be worked on. The topics of the AR program were about electrotherapy, phototherapy, ultrasounds, shock waves, and tecartherapy.

The students were informed and filled in the informed consent form before creating the control group and the experimental group, which would use the AR applications. A control and an experimental group were formed for both universities.

All students belonging to the experimental and control groups participated in the same lecture on electrotherapy, which was part of the second year of the Physiotherapy degree, both at the University of Cádiz and the University of Málaga. The experimental groups used AR applications to complement learning, integrating interactive 3D models that allowed the visualization of anatomical structures and simulations of electrode placement to apply electrotherapy procedures. At the University of Cádiz, the Aumentaty platform was used, while at the University of Málaga, Zapworks was used, although the content was the same in both cases, with the markers being adjusted to each technology. In addition, the applications provided step-by-step instructions for the correct configuration and application of electrotherapy.

In the experimental group, students received a lesson that included the use of AR applications. This complemented the subject content by integrating interactive elements such as links, videos, and 3D models. Students interacted with bookmarks that allowed them to access additional information, which enriched their learning experience.

Both applications used markers (physical or digital) to trigger AR experiences. They are compatible with mobile devices (iOS and Android), allowing users to interact with the content on their own phones. Both allow the visualization of 3D models and additional multimedia content (audio and text). They are used to enrich educational experiences. Nevertheless, there are some differences between Zapworks and Aumentaty; Zapworks is more versatile and advanced, with a visual interface that allows the creation of more customized and complex experiences, while Aumentaty is simpler and oriented to users with less technical experience. Zapworks relies on internet connection to access experiences through a browser or app, while Aumentaty can work offline. Aumentaty is more focused on the educational and cultural environments, with a more restricted use in terms of interactive possibilities, while Zapworks offers more flexibility to create different types of interactive experiences.

The control group received the same content via a traditional lesson without AR. The control group participated in a traditional lecture that consisted of a PowerPoint presentation covering the same content that was presented to the experimental group. This approach allowed for a direct comparison of the effectiveness of traditional teaching with AR-enriched teaching.

The data gathering procedure was carried out in the same way at both universities in order not to influence the results obtained. The System Usability Scale (SUS) [37] was used for this purpose. It is a methodological tool similar to the Likert Scale that measures the usability of an object, device, and application. The scale consists of 10 questions that can be scored from 1 to 5, where 1 means “Strongly disagree” and 5 means “Strongly agree” (Multimedia Appendix 1). First, the student was introduced to the two AR applications and was then asked a series of questions, which were the ones translated from the original test on Usability.gov [37]. The objective was to involve the students in the application of AR as well as to see the degree of agreement or disagreement with it in order to use it correctly and thus guarantee their agreement with the software and their correct learning.

A descriptive analysis of the control and the experimental group was conducted, considering the variables of age and sex. In addition, an individual analysis was conducted for the Rating and Usability variables in each of the Universities. In this analysis, the results of each population were obtained, and subsequently, analysis and interpretation of both variables were carried out together in order to contrast the previously mentioned hypotheses.

### Variables Studied

#### Demographic Variables

Participants were interviewed to obtain information about age and sex. These variables were obtained and analyzed separately according to whether the students belonged to the Universities of Málaga or Cádiz.

#### Rating Variable

The students’ academic performance was assessed by means of multiple-choice examinations at both the study sites. The rating variable considered the academic performance of the students participating in the study. These examinations were designed in a uniform manner to ensure comparability between the two programs, assessing the same key competencies and knowledge. The questions for the examinations were developed in conjunction with the teachers at each site, ensuring that the items reflected the established learning objectives. The grading of the examinations was performed in a standardized manner, using the same scoring criteria at both sites. The information was first analyzed and interpreted separately for the population of the University of Málaga and the University of Cádiz. This allowed for a direct comparative analysis of academic performance between the experimental and control groups.

#### Usability Variable

The Usability variable considered the use of AR applications in the populations of the Universities of Málaga and Cádiz. To evaluate the usability of AR applications, the SUS, a standard scale widely used in technology studies, was used. The SUS consists of 10 items that measure the perceived ease of use through a 5-point Likert scale, ranging from “Strongly Disagree” to “Strongly Agree” (Multimedia Appendix 1). The final score is obtained by summing the item responses, adjusting the values, and converting the result to a scale from 0 to 100.

In terms of its psychometric properties, the SUS has proven to be a reliable measure, with a Cronbach α coefficient typically exceeding 0.90, reflecting high internal consistency [38]. Its validity has been confirmed in a variety of studies and contexts, including educational settings, making it a suitable tool for the evaluation of the AR applications used in this study.

As with the rating variable, the analysis and interpretation were conducted individually for each population and then jointly using the Student *t* test and the Wilcoxon test. It should be noted that for the University of Cádiz, as the survey was anonymous, the date of birth and sex were used to assign the surveys to the ratings.

#### Usability and Rating Variables

Aiming to conduct an interpretation and analysis of the correlation between the Usability and Rating variables, the Spearman rank correlation coefficient was determined to establish the existence or absence of a correlation between variables.

### Statistical Analysis

The main aim was to compare the degree of usability of the two AR applications used during teaching. Analysis was performed using the R software (version 4.1.2; R Foundation for Statistical Computing). In addition, we also aimed to compare the academic performance of the groups using AR versus the control group and between the two experimental groups, which used two different AR applications.

It was accepted that the control group and the experimental group were composed of different people based on the sex and age variables determined by means of the contingency tables. As the dropout rate was 0% in the usability survey, owing to the same dates of birth, it was not possible to assign a value in the surveys and it was given as a lost value. Therefore, a total sample size of 199 participants was used for the design. First, the hypothesis (H0: μ1=μ2) of the existence of significant differences between the control group and the experimental group in academic performance was accepted as a general idea, given the significant differences in the variables of Rating and Usability due to the use of AR applications. Otherwise, the hypothesis (H1: μ1≠μ2) that there are no significant differences in academic performance due to the use of AR was assumed.

To evaluate the main hypothesis, the probability μ2 was set to the distribution μ1. In this case, the mean μ2 was within the 95% confidence limits, noting that the mean μ3 and μ4 were not within the limits. This indicated that there were significant differences at 95%.

For the analysis of the data obtained from the Rating and Usability variables, the data were checked for incompleteness, normality, and homoscedasticity. In the event that there was no normality or homoscedasticity, the pertinent analyses would be carried out for subsequent interpretation in order to be able to contrast the main hypothesis put forward.

An ANOVA with the Welch correction was performed for the variables age and rating. Two-by-two comparisons were performed using the Holm significance correction because it is the most robust with respect to homoscedasticity. In addition, comparisons of the ratings obtained by the four groups were performed using the Kruskal-Wallis test. For the statistical analysis of the Usability variable, the rating obtained and the usability survey (SUS) were used. A parametric T-Welch test analysis was performed for the Usability variable. Finally, a multiple regression analysis was conducted on the scores obtained by the participants, including sex and age as variables.

## Results

### Participant Characteristics

The initial sample consisted of 203 participants. However, 4 participants over the age of 30 years were excluded from the analysis to homogenize the study. The total sample analyzed was 199 participants. Out of the total number of participants, 103 belonged to the experimental group and therefore had access to the AR applications. A total of 49 students from the University of Cádiz used the Aumentaty AR artifact (ie, the Aumentaty group), and 54 students from the University of Málaga tested the Zapworks AR artifact (ie, the Zapworks group). A total of 96 participants belonged to the control group, of which 37 were from the University of Cádiz and 59 were from the University of Málaga.

### Regarding Demographic Variables

Regarding the population sample from the University of Cádiz, the mean (SD) age was 21.5 (1.2) years, with no significant difference between the control group (mean 21.4, SD 1.0) and the experimental group (mean 21.6, SD 1.3).

In the sample from the University of Málaga, there was a significant difference in the population, with a total mean (SD) age of 20.5 (1.2) years. The control group had a mean (SD) age of 20.9 (0.9) years, while the experimental group had a mean (SD) age of 20.2 (1.3) years. The demographic data are presented in Table 1.

**Table 1.** Demographic data (N=199).

Variable	Zapworks control group^a^ (n=59)	Aumentaty control group^b^ (n=37)	Zapworks group^a^ (n=54)	Aumentaty group^b^ (n=49)	*P* value*^c^*
Sex, n (%)					.40
Male	27 (46)	15 (41)	24 (44)	28 (57)	
Female	32 (54)	22 (59)	30 (56)	21 (43)	
Age (years)					<.001
Mean (SD)	20.9 (0.9)	21.4 (1.0)	20.2 (1.3)	21.6 (1.4)	
Median (IQR)	20.8 (20.3-21.2)	21.1 (20.8-22.0)	19.8 (19.5-20.5)	20.9 (20.6-22.4)	
Min, Max	19.2, 24.6	20.3, 25.7	19.0, 25.7	20.3, 26.3	

a Zapworks: The students of the experimental group at the University of Málaga used Zapworks augmented reality software.

b Aumentaty: The students of the experimental group at the University of Cádiz used the Aumentaty augmented reality software.

c Pearson *χ*2 test or Kruskal-Wallis rank sum test.

**Figure 1.**
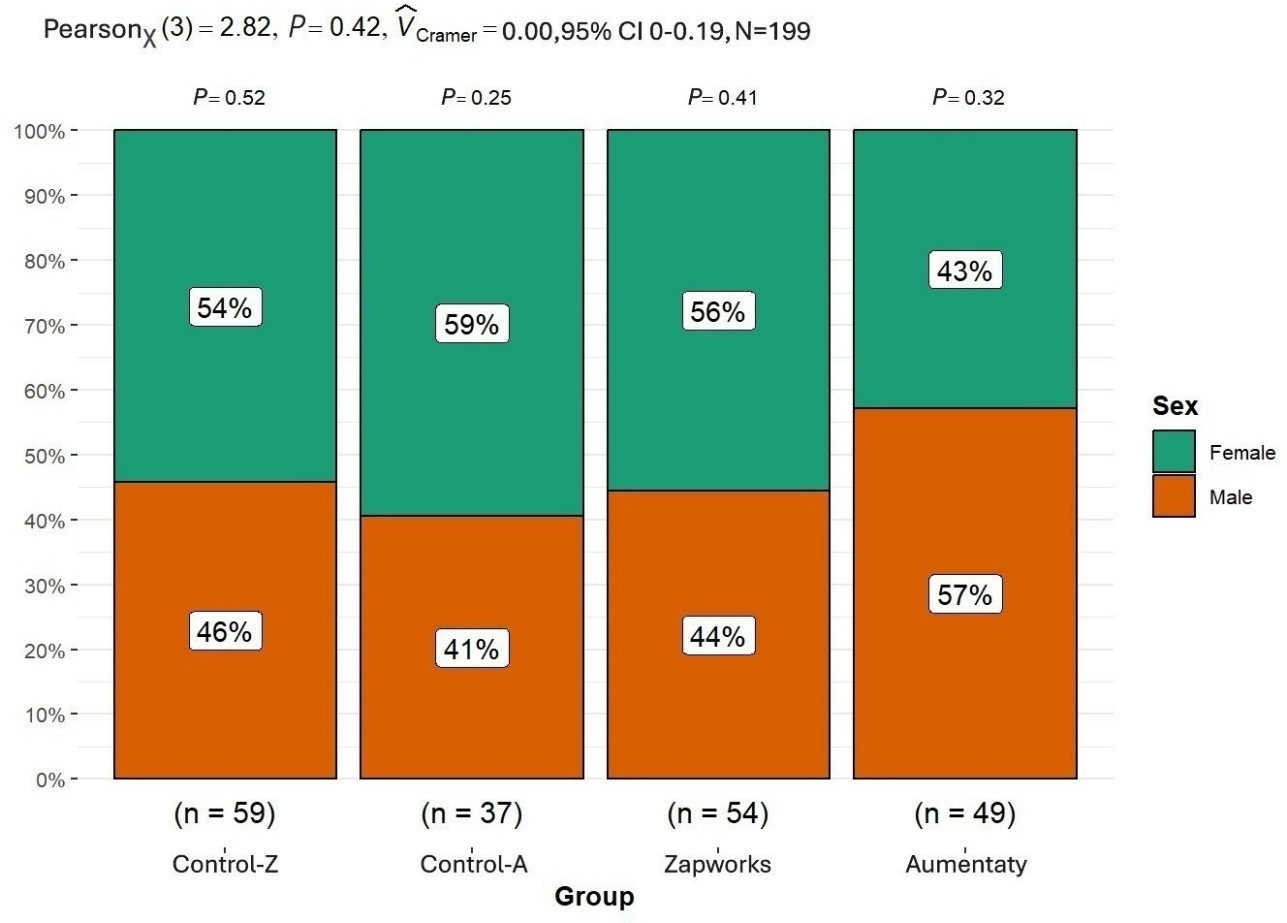
Sex difference in the distribution of participants across each group. Control-Z: Zapworks control group; Control-A: Aumentaty control group.

Figure 1 shows the distribution of the sexes according to the group, and there were no differences in the distribution of sex among the 4 groups. There were slightly more men in the Aumentaty group, but the Cramer V indicated that the effect size was very small.

### Regarding the Rating Variables

**Figure 2.**
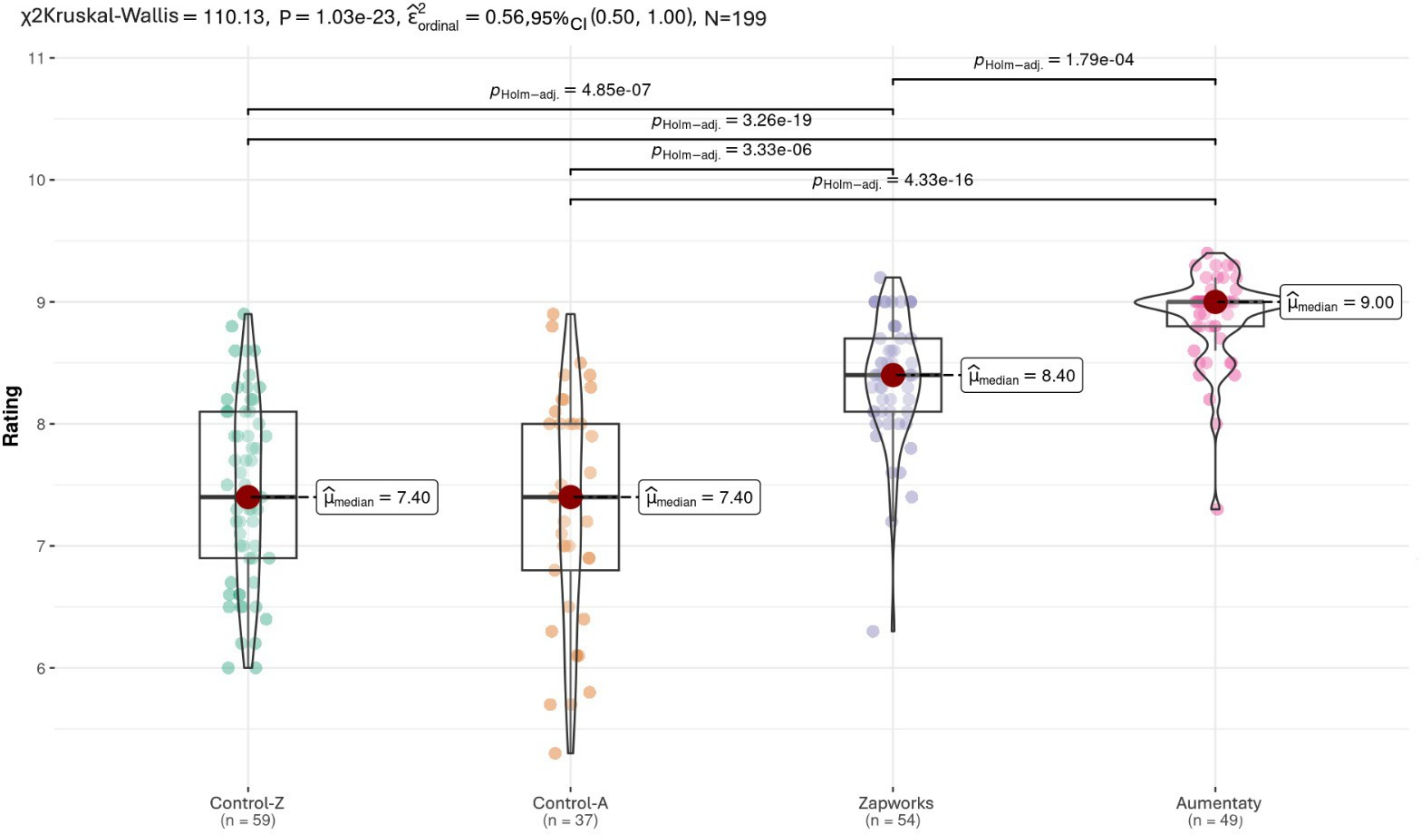
Comparing the data for the ratings variables. Control-Z: Zapworks control group; Control-A: Aumentaty control group.

The rating variables showed a significant difference individually in each of the samples in both Universities (*P*<.001), as presented in Table 2. In addition, Figure 2 shows the comparisons of the rating variables obtained by the 4 groups using the Kruskal-Wallis test. As shown in Figure 2, the ages of the control groups were similar, while the ages of both the control groups differed from those of the intervention groups. Additionally, the ages of the intervention groups differed from each other.

**Table 2.** Data for the rating variables.

Variable	N	Zapworks control group (n=59), median (IQR)	Aumentaty control group (n=37), median (IQR)	Zapworks group (n=54), median (IQR)	Aumentaty group (n=49), median (IQR)	*P* value^a^
Age (years)	199	20.86 (20.37-21.17)	21.06 (20.81-22.00)	19.83 (19.46-20.47)	20.99 (20.65-22.45)	<.001
Rating	199	7.40 (6.90-8.10)	7.40 (6.80-8.00)	8.40 (8.10-8.70)	9.00 (8.80-9.00)	<.001
Usability	93	N/A^b^	N/A	61.0 (59.0-64.0)	63.0 (60.0-64.0)	.049

a Kruskal-Wallis rank sum test.

b N/A: not applicable.

**Figure 3.**
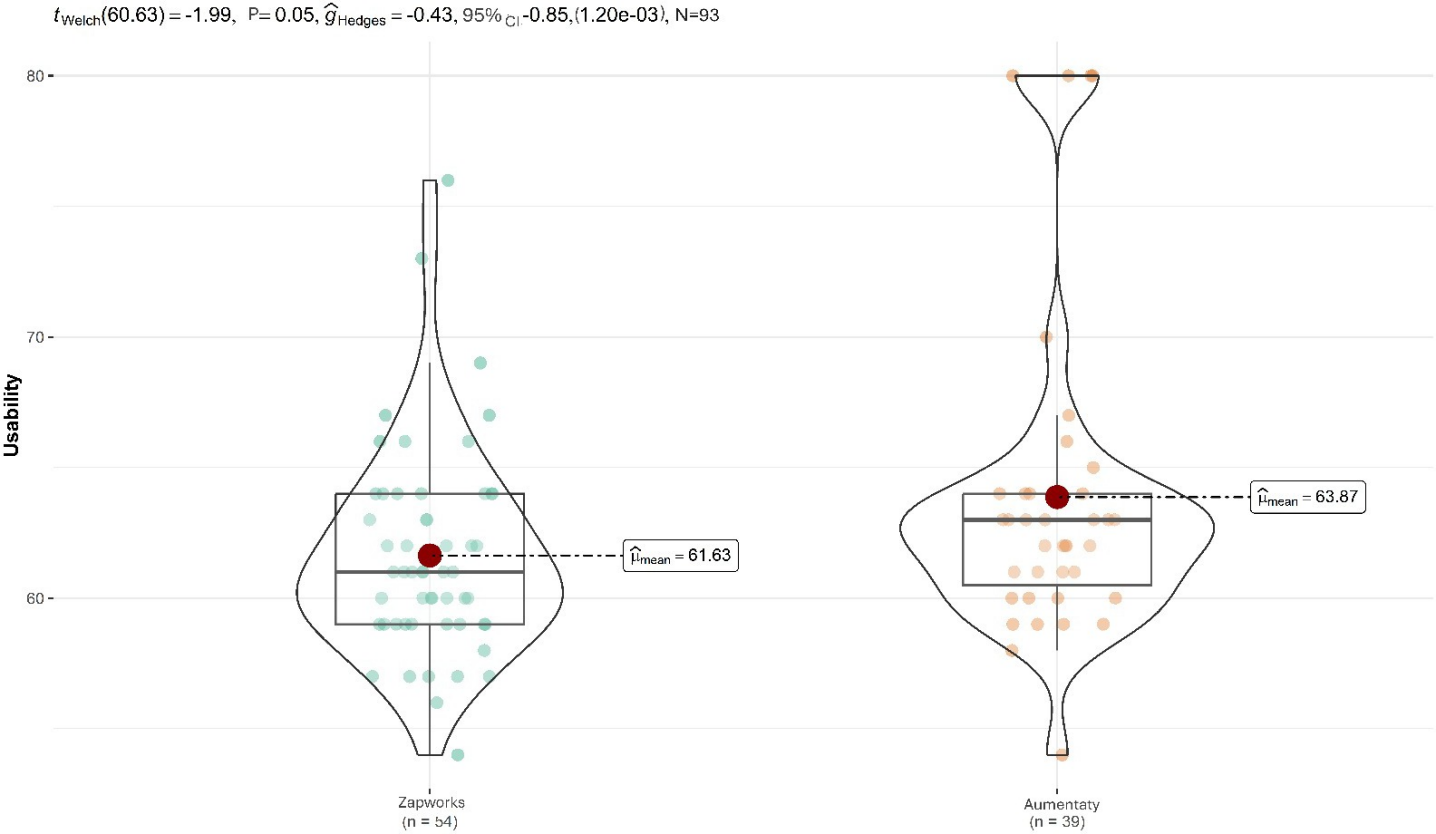
Univariate analysis of the usability variable.

Figure 3 shows the data on the difference between the Usability scores between groups. A moderate difference was observed between the University of Cádiz (mean 63.8, SD 6.15) and the University of Málaga (mean 61.6, SD 3.99), with a Hedges' g of 0.43. However, this difference was marginally non-significant (P=.05)

Finally, a multiple regression analysis was conducted on the scores obtained by the participants, including sex and age as variables. The results from both parametric and nonparametric analyses indicated that neither sex nor age had a significant effect on the participants’ scores. However, significant differences were found between students from the University of Málaga and the University of Cádiz (*P*<.001), with a beta coefficient of 1.4 favoring the Aumentaty group. Additionally, significant differences were observed between the control-A groups and the Zapworks group (*P*<.001), with a beta coefficient of 0.92 for the experimental group from Málaga, as presented in Table 3.

**Table 3.** Multiple regression analysis for the rating variables.

Characteristic	N	β	SE	95% CI	*P* value
Group					
Zapworks control	59	—^a^	—	—	—
Aumentaty control	37	−0.14	0.142	−0.42 to 0.15	.30
Zapworks	54	.92^b^	0.129	0.66 to 1.2	<.001
Aumentaty	49	1.4^b^	0.133	1.2 to 1.7	<.001
Sex					
Male	94	—	—	—	—
Female	105	.07	0.096	−0.12 to 0.26	.40
Age	199	.00	0.041	−0.08 to 0.08	>.99

a Not available.

b *P*<.001.

In Table 4, a multiple regression analysis was performed for the Usability variable, including the variable of sex. In this case, the differences between groups, which were on the borderline of significance, became more clearly significant. The Usability variable showed a significant difference (*P*=.03) in favor of the Aumentaty group as shown in Table 4.

**Table 4.** Multiple regression analysis for the Usability variable.

Characteristic	N	Beta	SE	95% CI	*P* value^a^
Group					
Zapworks	54	—^b^	—	—	—
Aumentaty	39	2.7	1.19	0.3 to 5.0	.02
Sex					
Male	46	—	—	—	—
Female	47	.66	1.06	−1.4 to 2.8	.50
Age	93	−.26	0.42	−1.1 to 0.6	.50

a *P*<.05.

b Not applicable.

**Figure 4.**
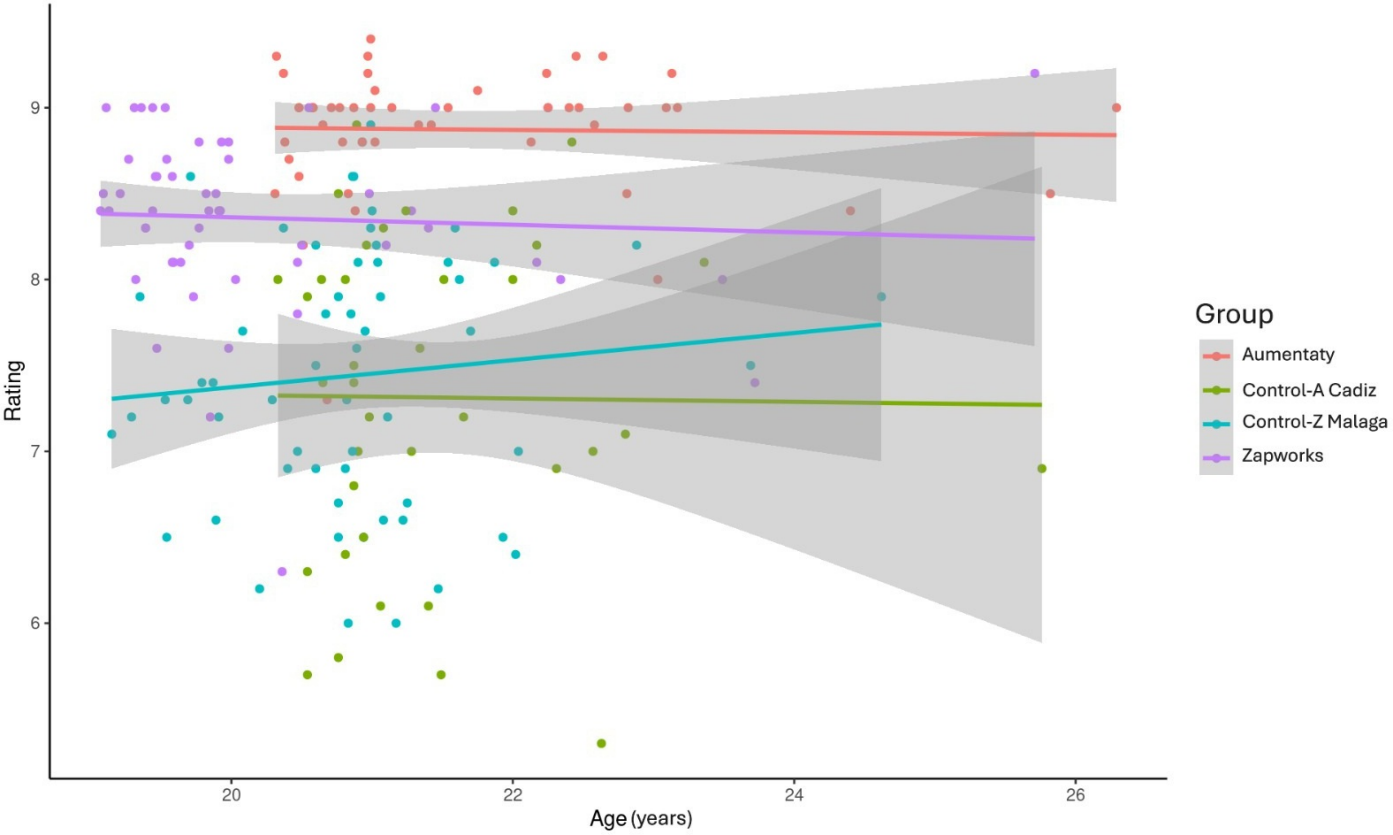
Relationship between age, group, and rating. Control-Z: Zapworks control group; Control-A: Aumentaty control group.

Figure 4 shows how age affects the scores; in terms of scores, age differences between individuals did not matter. A similar performance in scores was observed among participants from the control groups of both the universities. However, it should be noted that including students over the age of 26 years in the analysis might have influenced the scores obtained, depending on the AR technique used.

## Discussion

### Study Findings and Comparison With Previous Studies

The aim of this quasi-experimental design with the posttest was only to assess the use of two AR applications in two university populations and their influence on academic performance. It also sought to compare both populations in terms of the applications used and the academic performance.

The results of this study confirm the theoretical hypothesis that AR can enhance the learning experience compared to traditional methods. AR allows for greater interactivity with the content, which, as indicated by previous studies [39], can foster greater retention of information and increased student motivation. In contrast, the control group, who used traditional PowerPoint presentations, showed a more passive approach to learning, which supports previous research pointing to the limitations of traditional methodologies in capturing students’ continued interest [40].

From a theoretical point of view, comparison with the control group is fundamental to understand how AR changes the dynamics of learning. By offering a more immersive and participatory experience, AR can be aligned with constructivist theories of learning [41], which emphasize the active role of the learner in the construction of knowledge. This study provides evidence that AR not only enriches visual and contextual content, but can also alter the role of the learner within the learning process, making it more dynamic and proactive. However, it is important to note that the effectiveness of AR may depend on factors such as lesson design and students’ prior technological experience, which raises areas for future research.

When specifically examining the studies that utilized AR, positive results were found. First, the projection condition in physiotherapy education showed superior performance across various cognitive load dimensions compared to traditional teaching and tablet conditions; additionally, the use of AR and the projection of anatomical images resulted in a significant increase in the questionnaire score for the experimental group [42].

Second, significant differences were noted in students’ self-confidence regarding their learning abilities [39]. In another study, Kandasamy et al [43] implemented an AR application for teaching spine anatomy, which resulted in significant differences in students’ perceived comprehension and level of engagement [43].

Other studies have also yielded positive results in physiotherapy students; the researchers utilized a questionnaire based on the DeLone and McLeanís model [44] to evaluate the effectiveness of the educational experience. The main findings revealed that participants had a positive perception of the Augmented Studio experience, with high ratings in satisfaction, learner engagement, and system effectiveness [27].

In another study conducted by Pérez et al [28], the majority of students (89.8%) considered AR to be highly relevant in their educational processes. Regarding its usefulness and ease of use, 51.3% of respondents highlighted initial difficulties with familiarizing themselves with the AR app design and digital object creation. However, 82.1% viewed the incorporation of AR as highly positive for learning, finding it interesting (83.4%) and engaging, fostering active participation and reflection. Despite potential challenges, 79.5% of students recommended the use of AR and its apps as valuable resources for learning, promoting creativity and innovation [28]. Regarding the limitations of AR, a study conducted by Bacca et al [30] highlighted the significant challenge of maintaining superimposed information, particularly in marker-based AR applications; this limitation, reported at a rate of 9.3%, can result in student frustration and necessitates the need for improvements.

In a previous study about AR in anatomy learning, it was shown that there were significant differences (*P*<.0001) in the grades of students who belonged to the group that had used AR, showing an improvement in knowledge about anatomy [7]. This truly relevant fact is one of the results obtained in our design, where it was observed that the grades obtained in each sample (Universities of Málaga and Cádiz) were also significant (*P*<.001). These results showed a significant improvement in grades when AR is used in university teaching.

In a study similar to ours [31], which used Aumentaty with students from the University of Cádiz in the Physiotherapy degree, satisfactory results were observed in the improvement of knowledge comprehension, which translated into an improvement in the grades. The same happened in this study, when an interpretation of the Rating variable was conducted jointly for both universities through univariate and multiple regression analyses [31]. In the previous study, on the first univariate analysis for rating, the significant differences (*P*<.001) with a beta of 1.4 were observed in the sample from the University of Cádiz [31]. Furthermore, when comparing the control group with the Zapworks group between the two universities, the significant differences (*P*<.001) with a beta of .92 was observed, similar to the study where the significant results were in the group that used Aumentaty [31].

In another study that evaluated the use of AR in learning, a significant improvement in the visualization of the content was observed (*P*<.0001) in the group that had used AR. In addition, 3D compression, which is fundamental for the acquisition of knowledge in anatomy, was significantly greater in the experimental group with higher percentages compared to the control group [33]. Therefore, the significant differences were found in the group using AR and the results found were similar to the results (*P*<.001) observed in this study where an improvement in ratings was observed.

In a previous study where the usability was studied, the use of AR, one of the variables related to our design, was addressed [32]. The sample was taken from the participants who were obtaining a Degree in Nursing at the University of Valladolid, where 85% of the students who were familiar with AR believed that it offers many educational possibilities and, therefore, 86.67% thought that it could be implemented in the Faculty of Nursing [32]. Using these data, 93.33% responded that it would enhance their learning, which is why it is important to analyze and interpret the usability of this tool in Health Sciences students [32]. However, in our design, no significant differences were obtained (*P*=.11) in the degree of usability, which is why it was not possible to obtain conclusive facts about this variable.

### Limitations and Strengths

This design has a number of strengths. During the development of the quasi-experimental design, the sample size was adequate and there were similarities in the subject matter at both universities. The main limitation was that the control group received conventional teaching and therefore cannot be considered a pure control group.

### Conclusion

Significant differences are observed between the group using AR applications compared to the control group in both universities and in the two univariate contrasts. In the multiple regression analysis, which explains the greatest variability regarding the main effects, a greater effect is detected in the use of AR at the University of Cádiz compared to the University of Málaga. Finally, the results of the multiple regression analysis indicates that there are no effects of sex and age on the scores obtained by the participants in the study.

## Supplementary material

10.2196/54312 Multimedia Appendix 1 Questions from Usability.gov

## Abbreviations

AR: augmented reality
SPIRIT: Standard Protocol Items: Recommendations for Interventional Trials
SUS: System Usability Scale
