# Supplementary material for: Effectiveness of Augmented Reality in the Teaching of Health University Students: Quasi-Experimental Study
Source: JMIR Serious Games. 2025 Mar 27;13:e54312. doi: 10.2196/54312 (PMC11967754; doi:10.2196/54312)
Supplement: Multimedia Appendix 1 [file games-v13-e54312-s001.docx]

**Multimedia Appendix 1**

The following questions are the original ones, taken from the original test on Usability.gov:

| No. | Question | Scale | | | | |
| --- | --- | --- | --- | --- | --- | --- |
|  |  | Strongly disagree |  |  |  | Strongly agree |
| 1 | I think that I would like to use this system frequently | 1 | 2 | 3 | 4 | 5 |
| 2 | I found the system unnecessarily complex | 1 | 2 | 3 | 4 | 5 |
| 3 | I thought the system was easy to use | 1 | 2 | 3 | 4 | 5 |
| 4 | I think that I would need the support of a technical person to be able to use this system | 1 | 2 | 3 | 4 | 5 |
| 5 | I found the various functions in this system were well integrated | 1 | 2 | 3 | 4 | 5 |
| 6 | I thought there was too much inconsistency in this system | 1 | 2 | 3 | 4 | 5 |
| 7 | . I would imagine that most people would learn to use this system very quickly | 1 | 2 | 3 | 4 | 5 |
| 8 | I found the system very cumbersome to use | 1 | 2 | 3 | 4 | 5 |
| 9 | I felt very confident using the system | 1 | 2 | 3 | 4 | 5 |
| 10 | I needed to learn a lot of things before I could get going with this system | 1 | 2 | 3 | 4 | 5 |
